# MicroRNA-124 promotes hepatic triglyceride accumulation through targeting tribbles homolog 3

**DOI:** 10.1038/srep37170

**Published:** 2016-11-15

**Authors:** Xing Liu, Jiejie Zhao, Qi Liu, Xuelian Xiong, Zhijian Zhang, Yang Jiao, Xiaoying Li, Bin Liu, Yao Li, Yan Lu

**Affiliations:** 1 Department of Endocrine and Metabolic Diseases, Shanghai Institute of Endocrinology and Metabolism, Ruijin Hospital, Shanghai Jiao Tong University School of Medicine, Shanghai, China; 2 Department of Endocrinology and Diabetes, The First Affiliated Hospital of Xiamen University, Xiamen Diabetes Institute, Xiamen, China; 3 Department of Endocrinology, Fudan Institute for Metabolic Diseases, Zhongshan Hospital, Fudan University, Shanghai, China; 4 Department of Endocrinology and Metabolism, Shanghai General Hospital, Shanghai Jiaotong University School of Medicine, Shanghai, China; 5 Hubei Key Laboratory for Kidney Disease Pathogenesis and Intervention, Hubei Polytechnic University School of Medicine, Huangshi, Hubei, China; 6 Department of Laboratory Animal Science, Shanghai Jiao Tong University School of Medicine, Shanghai, China

## Abstract

An increase in hepatic triglyceride (TG) contents usually results in non-alcoholic fatty liver disease (NAFLD) and related metabolic diseases. However, the mechanisms underlying perturbations of hepatic TG homeostasis remain largely unknown. Here, we showed that MicroRNA-124 was up-regulated in the livers of C57BL/6 mice fed a short-term high-fat-diet (HFD). Adenoviral overexpression of miR-124 in C57BL/6 mice led to accumulation of excessive triglycerides and up-regulation of lipogenic genes in the liver. We further identified tribbles homolog 3 (*TRB3*) as a direct target of miR-124. AKT signaling, which is negatively regulated by TRB3, was enhanced by miR-124 overexpression. Moreover, restoration of TRB3 expression markedly abolished the effect of miR-124 on hepatic TG metabolism. Therefore, our findings revealed that miR-124 played a role in mediating high-fat-diet induced TG accumulation in the liver.

In mammals, liver is a metabolic organ that plays an important role in the regulation of triglyceride (TG) homeostasis. The homeostasis of hepatic TG metabolism is attributed to the precise regulation between TG acquisition and removal. Liver TG accumulates when an increase in de novo lipogenesis (DNL) or uptake of fatty acid cannot be offset by fatty acid oxidation or very low-density lipoprotein (VLDL) secretion^1^,^2^. Hepatic lipogenesis is primarily controlled by a transcription factor named sterol regulatory element binding protein 1c (SREBP-1c), which transcriptionally up-regulates most of lipogenic genes, including fatty acid synthase (*Fasn*), acetyl-CoA carboxylase (*ACC*) and stearoyl-CoA desaturase 1 (*SCD-1*)^3^. As a result, increased expression of SREBP-1c is closely associated with TG accumulation in the liver^4^,^5^. Of note, SREBP-1c expression can be regulated by multiple pathways, involving AKT, STAT3 and mTOR signaling^6^,^7^. Our previous studies demonstrated that SREBP-1c could also be regulated by a nuclear receptor cascade involving farnesoid X receptor (FXR) and small heterodimer partner (SHP) in obesity and aging^8^,^9^.

MicroRNAs (miRNAs), a class of small non-coding RNAs, regulate gene expression post-transcriptionally through binding to complementary regions in the 3′ untranslated regions (3′ UTRs) of target mRNA, which results in mRNA degradation or attenuated translation^10^,^11^. Several studies have shown that miRNAs may play a pathological role in the hepatic TG homeostasis. For instance, miR-122a-deficient mice developed hepatosteatosis as a consequence of dysfunctional expression of genes involved in TG synthesis, storage and secretion^12^,^13^. miR-33 affected TG metabolism in concert with their host genes *SREBF* through targeting key genes of fatty acid oxidation^14^,^15^. miR-378 could ameliorate hepatic steatosis in obese mice by directly targeting p110α subunit, a core component of insulin signaling^16^.

miR-124, which is abundantly expressed in the brain, plays a key role in neuronal differentiation and brain development^17^,^18^. miR-124 also participated in tumorigenesis by targeting oncogenic proteins including cyclin-dependent kinase 6 (CDK6), signal transducerand activator of transcription 3 (STAT3), rho-kinase2 (ROCK2) and enhancer of the zeste homologue 2 (EZH2)^19^,^20^,^21^. Consistently, downregulation of miR-124 was observed in several types of human cancers^20^,^21^,^22^,^23^,^24^,^25^. Moreover, miR-124 was shown to regulate pancreatic islet development through suppression of the forkhead box protein A2 (FoxA2) transcription factor and Rab27a^26^,^27^. However, the role of miR-124 in the regulation of hepatic TG homeostasis remains unknown.

In the present study, we identified a critical role of miR-124 in the regulation of SREBP-1c and hepatic TG homeostasis, which might help us to understand the pathogenesis of hepatic metabolic disorders.

## Results

### Short term high-fat-diet increases hepatic triglyceride contents in mice

**Figure 1:**
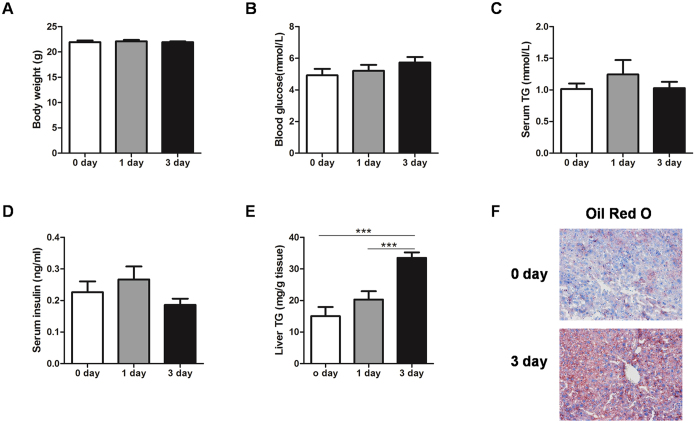
Short term HFD increases hepatic TG contents in mice. (**A–E**) Body weight (**A**), blood glucose (**B**), serum TG levels (**C**), insulin levels (**D**) and liver TG contents (**E**) in C57BL/6 mice fed a normal diet or high-fat-diet for 1 or 3 days. (**F**) Oil Red O staining showing TG accumulation in the liver. Original magnification, x200. ***P < 0.001.

Obese mice, such as *ob/ob* and *db/db* mice, and long-term high-fat-diet (HFD) fed mice, are usually used to investigate the mechanisms of hepatic TG homeostasis. However, these mice are characterized by many metabolic disorders, including obesity, insulin resistance and hyperglycemia, which may contribute as confounders to hepatic TG disorders^1^,^28^,^29^,^30^,^31^. Therefore, we employed short term (1 and 3 day) HFD mice to investigate hepatic TG homeostasis. As a result, there were no significant differences in body weight, blood glucose, serum TG or insulin levels among 0, 1 and 3 day HFD mice (Fig. 1A–D). However, hepatic TG contents were markedly increased in mice fed a HFD for 3 days (Fig. 1E), which was also confirmed by Oil Red O staining (Fig. 1F).

**Figure 2:**
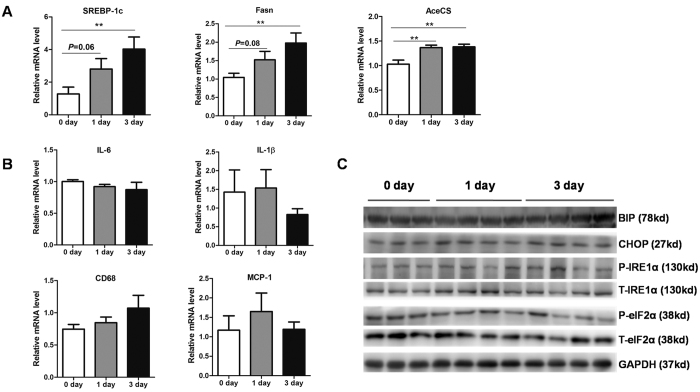
Short-term HFD promotes hepatic TG lipogenesis in mice. (**A**) Relative mRNA expression of lipogenic genes (*SREBP-1C, Fasn* and *AceCS*) in the liver from mice fed a HFD 3 days (n = 7–8). (**B**) Relative mRNA expression of pro-inflammatory cytokine (*IL 6, IL-1β, CD68* and *MCP-1)* in the liver from mice fed a HFD for 1 day or 3 days (n = 7–8). (**C**) Western blot analysis of key molecules (BIP, CHOP, p-IRE1α and p-eIF2α) in ER stress signaling in the liver from mice fed a HFD for 1 day or 3 days (n = 4). **P < 0.01.

To explore the molecular basis for hepatic TG accumulation, expression levels of those genes involved in TG homeostasis were determined by quantitative real-time PCR (qPCR) using livers of mice fed a HFD for 0 day, 1 day and 3 days. As a result, we found that lipogenic genes such as *SREBP-1c* and its down-stream target genes (*Fasn* and *AceCS*) were elevated in the livers of mice fed a HFD for 1 day and 3 days (Fig. 2A), suggesting that up-regulation of lipogenic genes contributed to hepatic TG accumulation in short-term HFD mice.

It has been well-established that inappropriate activation of inflammation and endoplasmic reticulum (ER) stress could promote hepatic TG accumulation in long term HFD obese mice^29^,^30^,^31^. Therefore, we assessed whether inflammation and ER stress were activated in livers of short-term HFD mice. mRNA levels of pro-inflammatory factors, including *IL-6*, *IL-1β, CD68* and *MCP-1* were not changed (Fig. 2B). Besides, protein levels of BIP, CHOP, phosphorylated IRE1α and eIF2α, markers of ER stress, remained unaffected (Fig. 2C). Taken together, our data indicate that the mechanism for hepatic TG accumulation and lipogenesis in short term HFD mice may be different from that in obese mice.

### miR-124 expression is elevated in short term HFD mice

**Figure 3:**
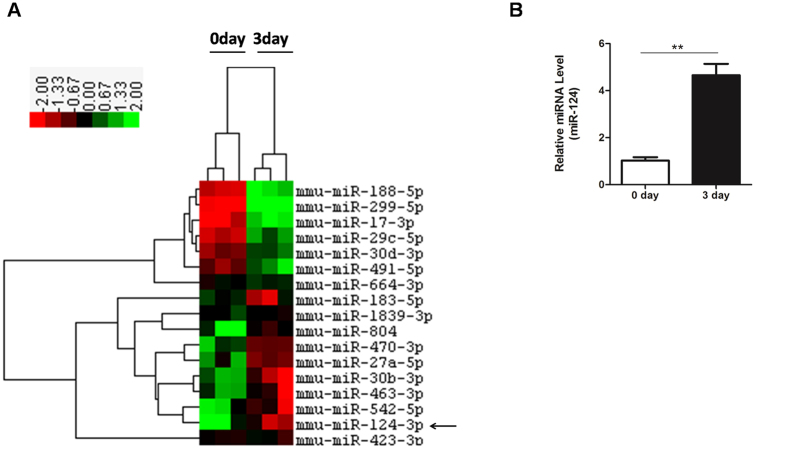
Up-regulation of miR-124 in short term HFD mice. (**A**) Cluster analysis of miRNA expression in livers from C57BL/6 mice aged 8 weeks fed with a normal diet (ND) or high-fat diet (HFD) for 3 days. Cluster in red indicates upregulation and in green indicates downregulation. (**B**) Comparative real-time PCR analysis of miR-124 expression in the liver of 3 days HFD mice. **P < 0.01.

In order to identify the factors that promote hepatic lipogenesis in short-term HFD mice, we performed a clustering analysis of MicroRNA arrays using livers of mice fed a normal diet or high-fat-diet for 3 days. Here, the time 0 day in the heat map represents normal diet (ND), while the time 3 day represents HFD for 3 days (Fig. 3A). As a result, we found that a number of miRNAs were significantly altered in the livers of mice of two groups (*P* < 0.05). Our screen revealed a pronounced up-regulation of miR-124 in the livers of 3 days HFD mice compared to ND mice (Fig. 3A), which was further confirmed by qPCR (Fig. 3B). Here, we focus on miR-124 because up-regulation of miR-124 was one of the most pronounced changes among differential expressed miRNAs (Fig. 3A). Besides, miR-124 has been shown play a critical role in neuronal differentiation, pancreatic islet development and tumorigenesis^17^,^18^,^20^,^21^,^22^,^23^,^24^,^25^,^26^,^27^. However, its role in the regulation of hepatic TG homeostasis remains uncharacterized. Therefore, miR-124 was chosen for further experiments.

### miR-124 promotes hepatic TG accumulation

**Figure 4:**
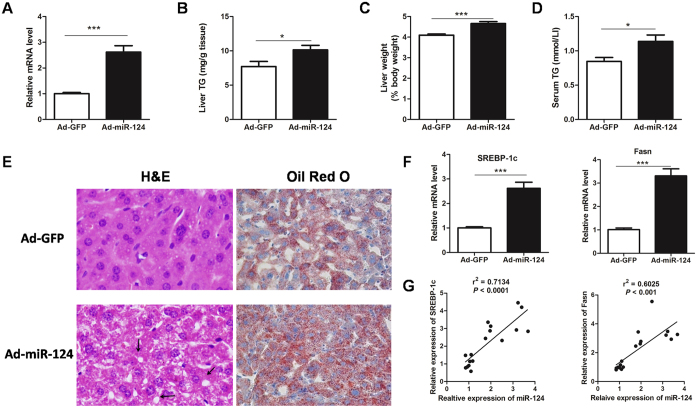
Overexpression of miR-124 increases hepatic lipogenesis. (**A**) The expression of miR-124 was determined in the liver of C57BL/6 mice infected with adenovirus (Ad-) containing GFP or miR-124 (n = 8–10). (**B–D**) Liver TG content (**B**) and weight (**C**) and serum TG levels (**D**) in mice infected with Ad-GFP Ad-miR-124 (n = 8–10). (**E**) Representative histology (H&E, left) or Oil Red O (right) staining showing TG accumulation in livers from mice injected with miR-124 versus GFP adenovirus. Original magnification, x200. (**F**) Relative expression of *SREBP-1c* and *Fasn* in two groups of mice. (**G**) Pearson R and P value for normalized *SREBP-1c* and *Fasn* mRNA levels versus miR-124 expression in livers of mice (n = 17). *P < 0.05, **P < 0.01, ***P < 0.001.

Next, to elucidate the role of miR-124 in the liver, adenovirus containing miR-124 or negative control (NC) were administered into C57BL/6 mice via tail vein injection. As shown in Fig. 4A, miR-124 expression levels were elevated in livers of mice infected with adenoviral miR-124 (Ad-miR-124). As expected, overexpression of miR-124 resulted in a marked increase in TG contents and liver weights (Fig. 4B,C), as well as serum TG levels (Fig. 4D). The observed alteration in hepatic TG content was further confirmed by H&E and Oil red O staining (Fig. 4E). However, the body weight, blood glucose, serum insulin and cholesterol levels were not changed (Fig. S1A–S1D). In agreement, mRNA levels of lipogenic genes, *SREBP-1C* and *Fasn,* were elevated and correlated well with the expression of miR-124 (Fig. 4F,G).

### TRB3 is a direct target of miR-124

**Figure 5:**
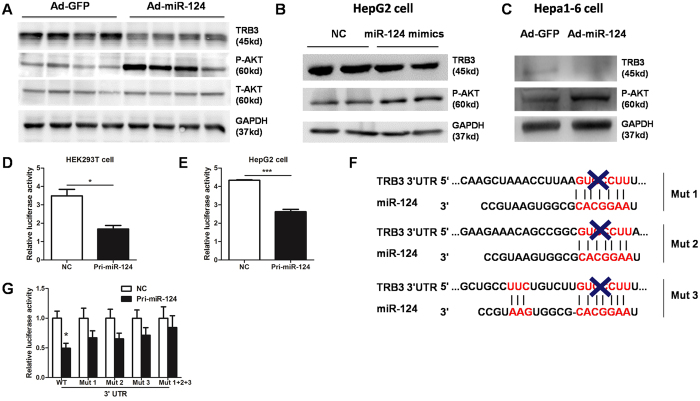
miR-124 regulates TRB3 expression. (**A**) Western blot analysis of TRB3 and phospho-Akt in the liver of mice infected with Ad-GFP or Ad-miR-124. (**B**) Western blot analysis of TRB3 and phospho-Akt in HepG2 cells treated with miR-124 mimics (100 nM) or vehicle control for 48 hours. (**C**) Western blot analysis of TRB3 and phospho-Akt in Hepa1-6 cells treated with Ad-GFP or Ad-miR-124 (100MOI) for 72 hours. (**D**,**E**) Relative luciferase activity of the firefly reporter containing the 3′UTR of TRB3 was detected in HEK293T cells (**D**) and HepG2 cells (**E**) co-transfected with pri-miR-124. (**F**) Sequence alignment of the 3′UTRs of *TRB3* and miR-124. (**G**) Relative luciferase activity of reporters containing either the wild-type or mutated 3′UTRs of *TRB3* gene (Mut1, Mut2, Mut3 and Mut1 + 2 + 3) were also analyzed in HEK293T cells. *P < 0.05, ***P < 0.001.

Using a stringent bioinformatics approach, we identified several putative murine miR-124 target genes, among which the gene encoding tribbles homolog 3 (*TRB3*) harboured a miR-124 binding site. Previous studies have shown that TRB3 is a pseudokinase that impaires insulin signaling by binding to Akt and blocking its activation^32^. Given that activation of Akt signaling could promote lipogenesis and hepatic TG accumulation^33^, we hypothesized that miR-124 may regulate TG metabolism through a TRB3-AKT pathway. As predicted, protein levels of TRB3 were decreased in the liver overexpressing miR-124 (Fig. 5A). Consistently, phosphorylation of Akt was increased as well (Fig. 5A). Overexpression of miR-124 also led to a decrease in TRB3 expression and an increase in Akt phosphorylation in HepG2 and Hepa1-6 cells (Fig. 5B,C).

To further determine whether TRB3 is a direct target gene of miR-124, we constructed a luciferase reporter containing the *TRB3* 3′UTR and co-transfected with pri-miR-124 or the control into HEK293T and HepG2 cells. As expected, miR-124 markedly repressed the luciferase reporter activity in both cells (Fig. 5D,E). Besides, there are three potential binding sites in the *TRB3* 3′UTR (Fig. 5F). Each mutation attenuated the reduction of luciferase activity by miR-124 (Fig. 5G), while mutation of all three binding sites resulted in abolished repression in luciferase activity (Fig. 5G). Thus, our data clearly indicate that TRB3 is a direct target gene of miR-124 in the liver.

### TRB3 restoration abolishes the effect of miR-124

**Figure 6:**
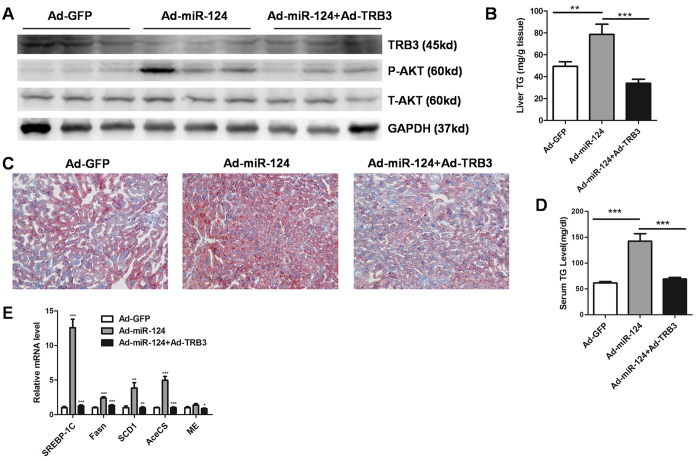
TRB3 overexpression alleviates miR-124 induced TG retention. (**A**) Western blot analysis of TRB3 and phospho-Akt in the liver of mice infected with Ad-miR-124 or both Ad-miR-124 and Ad-TRB3. (**B**) Liver TG levels in mice infected with Ad-miR-124 or both Ad-miR-124 and Ad-TRB3 (n = 8–9). (**C**) Oil Red O staining showing TG accumulation in the liver from mice infected with Ad-miR-124 or both Ad-miR-124 and Ad-TRB3. Original magnification, x200. (**D**) Serum TG levels in mice infected with Ad-miR-124 or both Ad-miR-124 and Ad-TRB3 (n = 8–9). (**E**) Relative mRNA expression of lipogenic genes in the liver from mice infected with Ad-miR-124 or both Ad-miR-124 and Ad-TRB3 (n = 8–9). *P < 0.05, **P < 0.01, ***P < 0.001.

To clarify whether TRB3 mediates the effect of miR-124 on hepatic lipogenesis, we restored TRB3 (Ad-TRB3) expression in the liver in which adenoviral miR-124 was overexpressed (Fig. 6A). As a result, TRB3 restoration completely ameliorated hepatic triglyceride accumulation (Fig. 6B,C), lowered serum TG levels and down-regulated hepatic lipogenic genes expression (Fig. 6D,E). Therefore, our data support the notion that miR-124 mediated TRB3 down-regulation plays a critical role in hepatic lipogenesis and TG accumulation.

## Discussion

In the current study, we for the first time identified miR-124 as a regulator in hepatic TG homeostasis. We demonstrated that miR-124 directly targeted TRB3, and led to activation of AKT and up-regulation of lipogenic genes. Thus, our findings suggest that manipulating miR-124 expression might provide an alternative approach for amelioration of hepatic TG accumulation.

At the molecular level, we functionally validate TRB3 as a miR-124 target. It has been shown that TRB3 inhibited insulin signaling by binding directly to Akt and blocking its activation^32^. Besides, specific overexpression or knockdown of TRB3 in livers promoted or improved insulin resistance, respectively^34^,^35^. However, whole-body deletion of TRB3 displayed normal hepatic insulin signaling and glucose homeostasis, including unchanged Akt activation^36^. Although the reason for this inconsistence remains unknown, we speculate that compensatory function of TRB3 in other metabolic tissues such as skeletal muscle and adipose tissues should be considered.

It has been well established that Akt is a critical node to promote lipogenesis^37^. This is supported by several evidences. First, Akt enhances post-translational processing of SREBP-1c through phosphorylating SREBP-1c precursor^38^. Second, Akt increases SREBP-1c activity via mammalian target of rapamycin complex 1 (mTORC1)^32^. Third, Akt prevents degradation of mature SREBP-1c through inhibiting glycogen synthase kinase 3 (GSK3), a kinase that promotes the proteasomal degradation of SREBP-1c^39^,^40^. Therefore, hepatic overexpression of constitutively active Akt induced marked steatosis and up-regulation of lipogenic genes, including *SREBP-1c, Fasn* and *SCD-1*^33^. Consistently, our data show that overexpression of miR-124 causes increased expression of SREBP1-c and TG accumulation in the liver. Of note, restoration of hepatic TRB3 expression almost completely abrogated the effect of miR-124 on lipids homeostasis, indicating that TRB3 might be the primary target of miR-124 in the regulation of liver TG metabolism.

Taken together, our study uncovers a novel miRNA, miR-124, that is responsible for early hepatic TG accumulation, preceding the systemic metabolic disorders. Our findings may expand our knowledge of mechanisms underlying the disturbance of hepatic TG metabolism and provide a promising therapeutic target for related diseases.

## Methods

### Animal experiments

Male C57BL/6 mice at age of 8–12 weeks were purchased from the Shanghai Laboratory Animal Company (SLAC, Shanghai, China). All mice were housed at 21 ± 1 °C with a humidity of 55 ± 10% and a12-hour light/12-hour dark cycle with free access to food and water. The high-fat diet (HFD) (D12492, Research Diets, New Brunswick, New Jersey, USA) contained 60% kcal from fat, 20% kcal from carbohydrate, and 20% kcal from protein. The normal diet (ND) contained 10% kcal from fat, 70% kcal from carbohydrate, and 20% kcal from protein. All animal protocols were approved by the Animal Care Committee of Shanghai Jiao Tong University School of Medicine and adhere to the criteria outlined in the “Guide for the Care and Use of Laboratory Animals”.

### Adenovirus preparation

Recombinant adenovirus expressing mouse miR-124 and TRB3 (Ad-miR-124, Ad-TRB3) was generated using the pAd-Easy system according to the manufacturer’s instructions. Viruses were diluted in PBS and administered via a tail vein injection using 2*10^9^ plaque-forming units per mouse.

### Microarray analysis

Total RNA was isolated from hepatic tissues using the standard TRIzol method according to the manufacturer’s instructions (Invitrogen, Shanghai). After having passed RNA quantity measurement using the NanoDrop2000, the samples were labeled using the miRCURY™ Hy3™/Hy5™ Power labeling kit (Exiqon, Vedbaek, Denmark) and hybridized on the miRCURY™ LNA Array (v.18.0, Exiqon). Following washing steps the slides were scanned using the Axon GenePix 4000B microarray scanner. Scanned images were then imported into GenePix Pro 6.0 software (Axon) for grid alignment and data extraction. Replicated miRNAs were averaged and miRNAs that intensities > = 30 in all samples were chosen for calculating normalization factor. Expressed data were normalized using the Median normalization. After normalization, significant differentially expressed miRNAs were identified through Volcano Plot filtering, which displays unstandardized signal (e.g. log(2) fold-change) against noise-adjusted/standardized signal (e.g. log(10) (p-value)).

### Hepatic and cellular TG measurement

Liver tissues (weighed ∼100 mg) were harvested and homogenized in chloroform/methanol (2:1 v/v) using a Polytron tissue grinder (Kinematica AG, Luzern, Switzerland). Lipid extracts were prepared by the classical Folch method. Extracts were dried under N2 flow and dissolved in isopropanol. Cellular TG contents were measured using commercial kits (Biovision, USA) according to the manufacturer’s instructions.

### Cell culture and luciferase assays

HepG2 and Hepa1-6 cell lines were purchased from the Cell Bank of Type Culture Collection, Chinese Academy of Sciences (CAS, Shanghai) and cultured in DMEM (Gibco, Shanghai, China) containing 10% FBS (Gibco), 100 IU/ml penicillin and 100 ug/ml streptomycin (Gibco). The primary miR-124 (pri-miR-124) sequence was amplified from the mouse genomic DNA and inserted into pcDNA3.1 vector (Invitrogen, USA). The complete 3′ UTR of murine *TRB3* containing either the wildtype or mutated miR-124 binding sites was cloned and inserted into pRL-null vector (Promega, USA). For luciferase assays, cells were co-transfected in 24-well plates in duplicate wells with TRB3 reporter vectors together with pri-miR-124 plasmids and empty vectors using Lipofectamine 2000 (Invitrogen, USA). Luciferase activities were measured consecutively by using the Dual Luciferase Reporter Assay System (Promega, USA).

### RNA isolation and real-time PCR

Total RNA of cells or tissues were extracted using standard TRIzol method according to the manufacturer’s instructions (Invitrogen, Shanghai). qRT-PCR was performed using a SYBR Green Premix Ex Taq (Takara, Japan) on Light Cycler480 (Roche, Switzerland). For miRNA detection, total RNA was reverse-transcribed using miScript II RT Kit (QIGEN, Shanghai). Subsequently, qRT-PCR was measured using miScript SYBR Green PCR Kit (QIGEN, Shanghai). U6 was used for normalization of miR-124 expression.

### Western blot

Hepatic tissues and cells prepared with radioimmunoprecipitation (RIPA) buffer containing 50 mM Tris-HCl (pH 8.0), 150 mM NaCl, 5 mM MgCl2, 2 mM EDTA, 1 mM NaF, 1% NP40 and 0.1% SDS, supplemented with protease and phosphatase inhibitors (Millipore, USA). 50 μg lysates were loaded onto 10% SDS-PAGE and transferred to polyvinylidenedifluoride (PVDF) membranes (Millipore, USA). The proteins were visualized with Immobilon Western Chemiluminescent HRP Substrate (Millipore, USA) according to the manufacturer’s protocol. Western blots were performed using antibodies against rabbit anti-p-AKT (Cell Signaling, #4060, 1:1000), rabbit anti-t-AKT (Cell Signaling, #9272, 1:2000), goat anti-TRB3 (Santa Cruz, sc-34211, 1:500), mouse anti-CHOP (Beyotime, #AC532, 1:1000), rabbit anti-BIP (Beyotime, #AB310, 1:1000), rabbit anti-p-eIF2α (Cell Signaling, #9721, 1:1000), rabbit anti-t-eIF2α (Cell Signaling, #9722, 1:1000), rabbit anti-p-IRE1α (Novus Biologicals, #NB100-2323, 1:1000), rabbit anti-t-IRE1α (Cell Signaling, #3294, 1:1000) and rabbit anti-GAPDH (Cell Signaling, #5174, 1:1000).

### Histological analysis

For hematoxylin and eosin staining (H&E staining), liver tissues were fixed in 10% neutral buffered formalin and embedded in paraffin. Sections were subjected to standard H&E staining. For oil red O staining, liver tissues were fixed in 4% paraformaldehyde in PBS, embedded in optimum cutting temperature compound (OCT), and cryosectioned. Frozen liver sections were stained with 0.15% oil red O according to standard procedures. Pictures were taken using an Olympus BX-51 microscope.

### Statistical analysis

All data were expressed as mean ± standard error of mean (SEM). Statistical differences were determined by a Student t test. Statistical significance is displayed as *p < 0.05, **p < 0.01 or ***p < 0.001.

## Additional Information

**How to cite this article**: Liu, X. *et al.* MicroRNA-124 promotes hepatic triglyceride accumulation through targeting tribbles homolog 3. *Sci. Rep.* **6**, 37170; doi: 10.1038/srep37170 (2016).

**Publisher’s note:** Springer Nature remains neutral with regard to jurisdictional claims in published maps and institutional affiliations.

## Supplementary Material

Supplementary Information
