# Supplementary material for: MicroRNA-124 promotes hepatic triglyceride accumulation through targeting tribbles homolog 3
Source: Sci Rep. 2016 Nov 15;6:37170. doi: 10.1038/srep37170 (PMC5109039; doi:10.1038/srep37170)
Supplement: Supplementary Information [file srep37170-s1.pdf]

MicroRNA-124 promotes hepatic triglyceride accumulation through targeting tribbles homolog 3

Xing Liu[1,+], Jiejie Zhao[1,+], Qi Liu[2,+], Xuelian Xiong[3], Zhijian Zhang[4], Yang Jiao[1], Xiaoying Li[1, 3], Bin Liu[5], Yao Li[6*], Yan Lu[1,3,5*]

[1]Department of Endocrine and Metabolic Diseases, Shanghai Institute of Endocrinology and Metabolism, Ruijin Hospital, Shanghai Jiao Tong University School of Medicine, Shanghai, China.

[2]Department of Endocrinology and Diabetes, The First Affiliated Hospital of Xiamen University, Xiamen Diabetes Institute, Xiamen, China.

[3]Department of Endocrinology, Fudan Institute for Metabolic Diseases, Zhongshan Hospital, Fudan University, Shanghai, China.

[4]Department of Endocrinology and Metabolism, Shanghai General Hospital, Shanghai Jiaotong University School of Medicine, Shanghai, China.

[5]Hubei Key Laboratory for Kidney Disease Pathogenesis and Intervention, Hubei Polytechnic University School of Medicine, Huangshi, Hubei, China

[6]Department of Laboratory Animal Science, Shanghai Jiao Tong University School of Medicine, Shanghai, China.

[+]These authors contributed equally to this work.

Correspondence and requests for materials should be addressed to Y.Li. (yao.li@shsmu.edu.cn) or Y.Lu. (lu.yan2@zs-hospital.sh.cn).

Supplementary Figure:

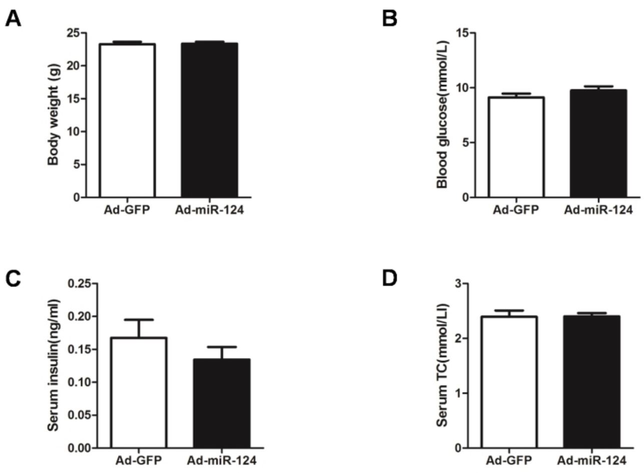

Fig.S1 Overexpression of miR-124 does not alter systemic metabolic parameters

(A-D) Body weight (A), blood glucose (B), serum insulin (C) and TC levels (D) in mice infected with Ad-GFP or Ad-miR-124 (n=8–10).
